# 

**Published:** 1851-02

**Authors:** 


*Buffalo Medical College.*—The annual commencement in this Institution will be on the last day of the term, Wednesday, the 26th inst. On the day preceding the commencement, the examination of candidates for graduation before the Board of Curators will take place. The address to the Graduates on commencement day will be delivered by Prof. Coventry. It is hoped that there will be a general attendance of the Curators, and that many other medical gentlemen interested in the prosperity of the College, will be present.

*To Publishers.*—We must ask the continued indulgence of those who have favored us with new publications. We have received several works which we shall notice so soon as we can find leisure to bestow upon them due examination.

*Surgical Report for the American Medical Association.*—The committee is invited to meet in the Charleston Hotel, South Carolina, the evening of the first Tuesday in May next. All professional brethren who have *surgical* facts connected with the improvement of this branch of tne profession during the year, will please address them to the chairman of the committee by the first of April, at Augusta, Georgia. As all cannot be reached by a circular, it is hoped no one will wait for a more direet application than this general invitation. PAUL F. EVE, M. D.

*Prof. of Surgery in the Louisville University, and Chairman of the Committe on Surgery of the Am. Med. Association.*

*Louisville, Ky., Dec.,* 1850.

---

*American Medecal Association.*—The Committee of Arrangements request all societies and other institutions anthorized to send delegates, to forward a correct list of those selected to attend the next annual meeting, to the Secretary, Dr. H. W. DeSaussure, at Charleston, S. C., on or before the first day of April.

In consequence of the resignation of Dr. Stille, one of the Secretaries, from ill health, all communications intended for the next meeting of the Association must be addressed to the remaining Secretary, Dr. H, W. De Saussure, Charleston, S. C.

The Fourth Annual Meeting of the American Medical Association will be held at Charleston, S. C., on the 2d Tuesday of May next.

Editors of Medical Journals will please give the above notices an early insertion in their respective Journals.—*Charleston Med. Journal.*

---

*To Correspondents.*—Communications from Drs. Hamilton, Gardner, and Hubbard will appear in our next No.

